# Mathematical modeling of the frozen zone dynamics: Towards using thermal imagers in cryotherapy

**DOI:** 10.1371/journal.pone.0313047

**Published:** 2026-04-16

**Authors:** Oleh V. Ivakhnenko, Olexandr F. Todrin, Vyacheslav Yu Globa, Mykola O. Chyzh, Gennadiy O. Kovalov, Sergey N. Shevchenko

**Affiliations:** 1 B.Verkin Institute for Low Temperature Physics and Engineering of the National Academy of Sciences of Ukraine, Kharkiv, Ukraine; 2 RIKEN Center for Quantum Computing, RIKEN, Wako, Saitama, Japan; 3 Institute for Problems of Cryobiology and Cryomedicine of the National Academy of Sciences of Ukraine, Kharkiv, Ukraine; Royal College of Surgeons in Ireland (RCSI), University of Medicine & Health Sciences, IRELAND

## Abstract

**Background:**

Thermal imaging is a convenient technique for cryoablation and cryotherapy monitoring; however, it does not provide insight into subsurface temperature distribution.

**Objectives and methods:**

Our mathematical model can predict a temperature penetration depth during cryotherapy based on surface thermal imaging. We also generalized this model to use with homogeneous media, such as hydrogels, and multilayered biological structures, including skin, muscle, and subcutaneous fat.

**Results:**

We developed a mathematical model to describe temperature dynamics in non-uniform materials with temperature-dependent thermodynamic properties and a phase-change boundary. We implemented the model with a graphical processing unit (GPU) to simulate the freezing behavior of hydrogels and biological tissues. The model was validated by comparing simulation results with experimental findings from hydrogel cryoapplications and previous in vivo studies on rats during the freezing phase.

Hydrogel, which exhibits thermodynamic properties like those of living tissues and possess optical transparency, enabled direct observation of the freezing front using visual morphometry. This model offers a practical tool for estimating optimal cryotherapy duration, helping to minimize damage to healthy cells. The position of the freezing front in semitransparent hydrogels was quantitatively assessed and found to be consistent with morphometric measurements.

**Conclusions:**

This study provides a useful framework for comparing in vitro and in vivo thermal field dynamics and for estimating optimal timing in cryoapplications. The mathematical model developed here, with its fast and efficient GPU-based implementation, can be extended to investigate thermal behavior in other uniform and non-uniform materials exhibiting temperature-dependent thermodynamic properties during freeze-thawing.

## 1 Introduction

In various applications of cryotherapy, such as cryodestruction and cryostimulation, mathematical modeling of non-stationary temperature fields provides recommendations for the precise planning of the cryoablation time [1]. Although cryotherapy remains largely empirical, modeling can predict and refine intuition by demonstrating different aspects of dynamics in living tissue, see Refs. [2–4] for review. Various theoretical aspects of modeling have been studied before, such as freeze-thaw cryosurgical cycles [5], estimation of the stable frozen zone volume [6], thermal stresses appearing around a cryoprobe [7].

It is often more practical and ethical to use biological tissue gel phantoms instead of experimenting with living tissues [8,9]. This approach allows for the utilization of the transparency of the hydrogel to observe the ice boundary position dynamics [10] and compare it with numerical calculations. In addition, hydrogels have consistent thermodynamic properties: therefore, there is no need to collect massive statistics from the experiments.

Cryoapplication assumes movement of the freezing front, which is described by a Stefan-type free-boundary problem [3,11,12]. Mathematical modeling of thermal field dynamics in biological tissue should also consider body heating to describe the metabolic heat generation and blood perfusion [13,14].

To perform cryosurgical procedures, the extent of freezing should be monitored precisely [2]. While local measurement techniques can be applied, such as inserting a thermal sensor, it usually disrupts the temperature field and leads to additional damage to the soft tissues. So, different techniques can be used including electrical impedance tomography, X-ray computed tomography, magnetic resonance imaging, and ultrasound imaging. The rarely used approach exploits thermal imagers, which provide an accessible and convenient tool [15,16]. Thermal imagers can be used for monitoring even low temperatures [17], which makes them helpful for cryotherapy [18]. One of the problems that prevents the widespread use of thermal imagers for cryotherapy is that they provide only surface thermal-field images and do not provide information about temperature distribution in bulk. We hope that this can be addressed by developing convenient modeling, to which we devote our present study.

Previously, for mathematical modeling, it was assumed that the thermodynamic parameters were not temperature dependent [1,5,19,20] mainly due to a lack of data, difficulties in computations, and the impossibility of finding an accurate analytical solution with temperature-dependent parameters. Here, we developed a mathematical method that considers the temperature dependence of the thermodynamic parameters, which increases the accuracy of the modeling, because thermodynamic parameters vary meaningfully within a wide temperature range during cryoablation.

## 2 Formulation of the problem

To simulate the impact of the cryoapplicator on biological tissue or hydrogel, we solve the heat equation. For clarity, we will consider a hydrogel, but the same model can be applied to biological tissues. In this study, we considered the axial symmetry case to eliminate the third dimension and considered only the radius and depth in our temperature field simulation [13,19–21].

### 2.1 Using hydrogel for temperature dynamics of living tissues

Hydrogel is commonly used as a model media to investigate different mechanical and thermodynamic features of the living tissues since many features of these materials are quite similar [8,9].

#### 2.1.1 Main features of biological tissues and hydrogel.

Hydrogels and biological tissues contain solutions that typically do not have a fixed freezing temperature. The solution usually freezes within a certain temperature range, for example, from –0.1°C to –14°C for 5% gelatin hydrogel [8].

In addition, the latent heat of phase change is distributed over this range of temperatures. Another feature of the hydrogel and the biological tissues is the noteworthy temperature dependence of the thermodynamic parameters in so wide temperature range (from –196°C to 34°C). Despite the more complicated multilayer morphological structure of the living tissues, cryoapplication results can be very close to hydrogel because of the large cooling power of the cryoapplicator, fast freezing, and large temperature gradient. This fast freezing can be disordered by a major vessel nearby with high blood flow, but as demonstrated in Ref. [21] the effect of the major blood vessel can also be considered in the experiments on the hydrogel.

#### 2.1.2 Limitations.

There are also several limitations of the hydrogel to achieve exact quantitative agreement, such as: 1. lower initial temperature, due to liquefaction temperature of the gelatin hydrogel which is 34°C which is lower than the typical body temperature of mammals; 2. hydrogel shows quantitatively good coincidence of thermodynamic evolution to multilayered living tissues with layers that have similar thermodynamic properties. For example, the impact of a significant layer of subcutaneous fat will lead to significantly different results of cryoapplication, as we discuss in more detail in Sec. 3.2.1.

#### 2.1.3 Mathematical modeling for cryosurgery.

So, “to improve cryosurgery further, there is the need to develop mathematical cryosurgery optimization techniques” [2]. To this end, we present mathematical modeling of freeze- thawing in both a biological tissue and a model medium gel-phantom. In doing so, we discuss modern calculation capabilities, such as using a GPU, validate our calculations by comparing with measurements, and make conclusions on using thermal imaging for estimating the freezing volume and temperature field distribution inside the tissue or hydrogel. This research was aimed to theoretically predict the dynamics of the temperature field in the depth of an object based on the temperature on its surface with phase transitions and temperature-dependent parameters during cryotherapy.

#### 2.1.4 Contents.

Accordingly, the rest of the paper is organized as follows. In Sec. 2 we present basic equations for the free-boundary Stefan-like problem. Details of the calculations, such as the finite-difference method and heat flow continuity condition, are presented in Appendix A. Our main results, which describe the underlying physics, are presented in Sec. 3. Solutions and explanations for the hydrogel and living tissue are presented in Secs. 3.1 and 3.2 with detailed parameters presented in Appendix B. The solution of the heat equation using a GPU is described in detail in Appendix C. In Section 3.3 we describe our experimental observations of ice formation in the hydrogel. Section 4 is devoted to the discussion of the obtained theoretical and experimental results. Finally, in the Conclusions section, we sum up our main results and discuss further ways to improve this technique.

## 3 Materials and methods

### 3.1 Heat equation

The thermal conductivity equation describes the energy flux *q* through a material, which is proportional to the temperature gradient [22]:


q(r,t)=−k∇T(r,z,t), (1)

where *k* is the thermal conductivity, *T* is the temperature, and ∇ denotes the scalar temperature field gradient.

To model the dynamics of heat distribution, we apply the heat equation, which governs the spatial and temporal distribution of temperature:


$$\frac{\partial T}{\partial t}=\alpha \nabla^{2}T(r,z,t).$$ (2)

The thermal diffusivity coefficient *α* is defined as:


$$\alpha =\frac{k}{\rho C_{\text{p}}},$$ (3)

where *C*_p_ is the thermal capacity for constant pressure, *ρ* is the density, $\nabla^{2}$ is the Laplace operator. To improve the accuracy of thermal modeling, we consider the temperature dependence of all thermodynamic parameters. This leads to a non-uniform heat equation of the form as follows [23]


$$\frac{\partial T}{\partial t}=\frac{1}{\rho (T)C_{\text{p}}(T)}\nabla \left(k(T)\nabla T(r,z,t)\right).$$ (4)

### 3.2 Stefan problem

Problems involving dynamic phase-change boundaries are typically formulated as Stefan ones, Ref. [14]. Here changing the phase boundary position depends on the energy flow through the boundary. In such cases, the position of the phase boundary evolves based on the energy flux across it. The energy flux is defined as the difference between incoming and outgoing thermal energies at the phase-change interface. For a one-dimensional flat geometry, the Stefan condition can be expressed as


$$\left[k_{1}\frac{\partial T_{1}(z,t)}{\partial z}-k_{2}\frac{\partial T_{2}(z,t)}{\partial z}\right]=\lambda \rho \Delta \xi ,$$ (5)

where *k*_1,2_ denotes the thermal conductivity coefficients of phases 1 and 2, and *T*_1,2_ is the temperature of the respective phase, *z* is the spatial coordinate. The term *λ* refers to the latent heat of melting, *ρ* is the density of the material undergoing phase change, and Δξ is the volume of material transitioning at the boundary. This volume change can be positive or negative depending on the energy flux at the phase-change interface, resulting in either advancement or downturn of the boundary position.

In our analysis, we assume a simplified formulation of the Stefan problem by applying the continuous heat equation Eq. (4), where the latent heat of phase change is incorporated into the effective heat capacity, as detailed in Appendix B. This method is particularly suitable for systems in which latent heat is distributed over a broad temperature range and no distinct phase transition temperature exists [24,25].

### 3.3 Geometry and boundary conditions

**Fig 1:**
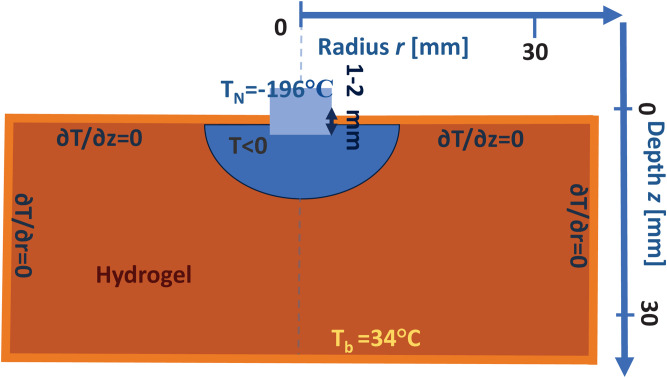
Diagrammatic representation of the cylindrical geometry used for simulation of hydrogel freeze-thawing, along with the initial and boundary conditions applied in the model.

Fig 1 illustrates the principal layout of the cryo-application in a two-dimensional cylindrical geometry with axial symmetry. In this setup, a cryoapplicator—maintained at a temperature near the boiling point of liquid nitrogen—is pressed 1–2 mm into the hydrogel. We assume that the typical size of the frozen region is notably smaller than the overall dimensions of the hydrogel sample.

In this study, the cryoapplicator has a tip radius of *r*_ap_ = 4 mm. For radial coordinates *r* < *r*_ap_, the temperature is held constant at $T_{\text{N}}=-196^{\circ }C$ approximating the boiling temperature of liquid nitrogen. This condition is kept throughout the freezing phase. During the subsequent thawing phase, a boundary condition is applied at the depth coordinate *z* = 0 where the heat flux is set to zero—indicating no thermal exchange across the surface at zero depth.

1.1. To simulate a more realistic scenario, we also consider the cryoapplicator being slightly pressed into the hydrogel. Specifically, we assume that the initial temperature within the region defined by the cryoapplicator tip radius *r*_ap_ and depth *z*_ap_ = 1 mm is equal to the temperature of the cryoapplicator *T*_N_. Additionally, we incorporate a heat source located deep within the sample by introducing a thermal bath at the maximum simulation depth *z* = *z*_max_ maintained at a temperature $T_{\text{b}}=34^{\circ }$C. This boundary condition reflects the heat flow from deeper tissue layers in biological systems. The value of *z*_max_ speaks for the lower boundary of the computational domain used in our numerical simulations.

1.2. The cryoapplicator typically lacks thermal insulation on its lateral surfaces, allowing heat from the hydrogel surface to dissipate into the surrounding air. This results in a larger freezing area on the skin or hydrogel compared to simulations that neglect air conductivity. However, this extra freezing effect due to air conduction influences only a superficial layer and dissipates rapidly once the cryoapplicator is removed. Therefore, we account for the thermal conductivity of air near the sample surface in our model.

2.1. The boundary condition at a depth far beyond the freezing region assumes a constant temperature of $T_{\text{b}}=34^{\circ }$C. The initial condition at the phase boundary position *r* > *r*_ap_ also assumes a constant temperature equal to *T*_b_.

2.2. The boundary condition at the cryoapplicator interface assumes a constant temperature *T*_N_, based on the premise that the cryoapplicator’s cooling capacity significantly exceeds the incoming heat flux.

3. In our calculations after removing the cryoapplicator, its volume is replaced with air. During the thawing stage, we assume no heat exchange occurs between the sample and the surrounding air.

Summary of boundary and initial conditions for the freezing stage:

- The cryoapplicator maintains a constant temperature of $T_{\text{N}}=-196^{\circ }$C approximating the boiling point of liquid nitrogen;
- The initial temperature of the sample is $T_{\text{b}}=34^{\circ }$C;
- The temperature at the lower boundary remains constant at $T_{\text{b}}=34^{\circ }$C;
- No heat transfer occurs through the side boundaries;

$$\left\{ \begin{array}{c} T=T_{\text{N}}{|}_{r⩽r_{\text{ap}},\;z<z_{\text{ap}}}\\ T=T_{\text{b}}{|}_{r>r_{\text{ap}},\;z>z_{\text{ap}},\;t=0}\\ T=T_{\text{b}}{|}_{z=z_{\text{max}}}\\ \frac{\partial T}{\partial r}=0{|}_{r=r_{\text{max}}} \end{array}\right.$$ (6)

Thawing-stage boundary conditions:

- Heat transfer through the sample–air interface is neglected;
- Deep within the sample, the temperature is maintained at a constant value of *T*_b_;

$$\left\{ \begin{array}{c} \frac{\partial T}{\partial r}=0{|}_{r=r_{\text{max}}}\\ \frac{\partial T}{\partial r}=0{|}_{r⩽r_{\text{ap}},\;z=z_{\text{ap}}}\\ \frac{\partial T}{\partial r}=0{|}_{r=r_{\text{ap}},\;z⩽z_{\text{ap}}}\\ T=T_{\text{b}}{|}_{z=z_{\text{max}}} \end{array}\right.$$ (7)

### 3.4 Numerical simulation

The heat dynamics challenge presented here involves a large temperature gradient and a moving phase-change boundary within a complex geometry. Such problems are typically unsolvable by analytical methods [26]. Therefore, we employ the finite-difference method to solve the heat equation numerically [27,28]. A detailed description of this method is provided in Appendix A.

Considering the specific characteristics of hydrogels and living tissues—such as phase transitions occurring over a temperature range and thermodynamic parameters that are highly temperature-dependent—we solve the heat equation using temperature-dependent coefficients throughout the simulation domain. The model incorporates the latent heat of phase change as an additional heat capacity within the freezing temperature range. In most cryoablation simulations, thermodynamic parameters for each phase are assumed to be temperature-independent [2–6] primarily due to the lack of detailed data across varying temperatures. However, these parameters can vary drastically over the wide temperature range encountered in cryo-ablation from the extremely low temperatures of liquid nitrogen to physiological body temperatures. Incorporating temperature-dependent thermodynamic properties enhances the accuracy of the mathematical model.

Such a model can be valuable for predicting the size of specific isotherms under controlled conditions in biological tissues, and other materials with known thermodynamic properties. Nevertheless, experimental results may differ due to various influencing factors, although the overall qualitative behavior of the thermal dynamics should remain consistent.

### 3.5 Assumptions in our mathematical model

Several assumptions have been made in the development of our mathematical model, primarily to simplify the problem and to omit various complex processes that occur during experiments:

- Lateral boundaries: We assume no heat transfer through the sides of the simulated volume. This implies that the freezing front remains sufficiently distant from the side boundaries, resulting in an extremely small temperature gradient in those regions.
- Depth boundary: A similar assumption is applied to the depth coordinate. We assume that the freezing front does not approach the lower boundary of the simulated domain.
- Spatial resolution: A sufficiently small grid step size is required in the finite-difference method to accurately resolve temperature dynamics. For example, the grid steps along the radial coordinate *r* and the depth coordinate *z* should be comparable to the smallest structural features being simulated—or even smaller, by an integer factor—to enhance simulation accuracy.
- Temporal resolution: The time step Δt must be small enough to ensure accuracy in each finite-difference method time increment [29], especially given the steep temperature gradients that arise during cryoapplication. Additionally, the time step must be adequately small to maintain numerical stability and to minimize errors in calculating the effective thermal capacity, which exhibits a sharp peak near the cryoscopic temperature *T*_f_.
- Thermodynamic parameters: We assume that the temperature-dependent thermodynamic parameters and the polynomial approximations used in our calculations (described in Appendix B) are sufficiently accurate. This is particularly relevant for temperatures below –150°C for hydrogels [8] and below –50°C for biological tissues. These thresholds typically represent the lower limits of available experimental data on thermodynamic properties [30–36]. Consequently, at lower temperatures, the approximated parameters used in the simulations may deviate from actual values.
- In biological systems, the thickness of tissue layers can vary significantly between samples and across individual animals. For example, in Section 3.2.2 we discuss the impact of varying subcutaneous fat thickness on cryoablation outcomes. Additionally, thermodynamic parameters within the same tissue type may differ; however, as shown in Section 3.1, such alterations do not greatly affect the overall temperature dynamics.
- Due to the lack of thermal insulation on the sides of the cryoapplicator, thermal convection occurs through the surrounding air, leading to additional cooling of the sample surface and an increased radius of the frozen area. In our model, this effect is simplified as an additional surface cooling term. While this simplification may reduce model accuracy, it allows for a more tractable simulation. A detailed discussion is provided in Appendix A.5.

### 3.6 Experimental materials and methods

To validate the theoretical calculations described above, we conducted *in vitro* experiments to study the formation and progress of an ice hemiellipsoid *in vitro*. A 5% gelatin hydrogel was used as the phantom material, and a homemade copper cryoapplicator actively cooled with liquid nitrogen (radius 4 mm) served as the freezing source. The radius and depth of the ice hemi-ellipsoid formed beneath the cryoapplicator were determined by means of visual morphometry, based on video recordings of the freeze-thawing processes. These recordings were analyzed using the AxioVision 4.7 software. Details of the measurement setup, gel phantom preparation, cryoprobe design, and camera configuration as well as comprehensive experimental data on isotherm dynamics (0° C, –20° C, –40° C) during prolonged low-temperature exposure with a flat applicator have been recently published in Ref. [21]. In this study, we present visual morphometry results obtained by observing the shape of the ice spot within a transparent hydrogel.

Gel phantom preparation: gelatin granules (5% by weight) were soaked in distilled water at room temperature (20 ± 2°C) for 2 hours. The mixture was then heated to 60°C to achieve a homogeneous solution, which was subsequently left to swell at room temperature for 20–22 hours, until complete swelling.

The hydrogel used in all experiments was based on gelatin hydrolysate of enzymatic origin (Sigma–Aldrich, catalog no. G0262). According to the manufacturer, the material corresponds to gelatin hydrolysate (CAS no. 91079-43-5; EC no. 293-431-0).

Experimental setup: a custom multichannel measurement setup was developed to analyze freeze-thawing processes *in vitro* [21]. The setup included:

- A container with the gelatin phantom;
- A liquid nitrogen-cooled cryoapplicator;
- Resistance thermometers connected to a multichannel analog-to-digital signal converter;
- A low-temperature thermal field analyzer with dedicated software;
- A personal computer for visualization and automatic data recording;
- The cryoinstrument used was a homemade device featuring a flat copper 4.0 mm in diameter applicator cooled with liquid nitrogen. The operating mode of the cryoinstrument was verified by measuring the temperature of its working surface, which ranged from −192.0°C to −194.8°C.

Experimental procedure: the gel phantom (initial temperature: 20 ± 2°C) was placed in a container immersed in the one filled with liquid at the same temperature. A circulation thermostat with a pump maintained the liquid temperature by continuously moving it. Resistance thermometers were positioned at designated points on the gel phantom using a fixing device and connected to the signal converter. A thermal imager was mounted on a tripod at a fixed angle and distance from the phantom surface. Video cameras were arranged to capture multiple viewing angles of the cryo-exposure zone. After testing the equipment and recording initial data, liquid nitrogen was introduced into the cryoapplicator mounted on a height-adjustable tripod. Once the applicator surface reached the target temperature, the gel phantom was exposed to low temperatures. At the same time, thermographic imaging, direct thermometry, and video recording of the freeze-thawing were performed [21].

## 4 Results

### 4.1 Frozen-zone dynamics in hydrogel

Hydrogel serves as an excellent model medium for studying the effects of freezing in biological tissues due to their thermodynamic properties, which are close to those of living tissues—primarily because of similar moisture content [8,9]. This resemblance enables strong alignment between mathematical modeling and experimental results in biological systems [16,21]. One key advantage of hydrogel is the ease of measuring thermodynamic parameters across a wide temperature range, as detailed in Ref. [8]. Another important benefit is the consistency of these parameters, which allows for highly reproducible cryoapplicator impact simulations and facilitates precise theoretical and numerical adjustments. In contrast, biological tissues exhibit great variability in thermodynamic properties due to differences in composition and hydration levels, which can vary from sample to sample.

**Fig 2:**
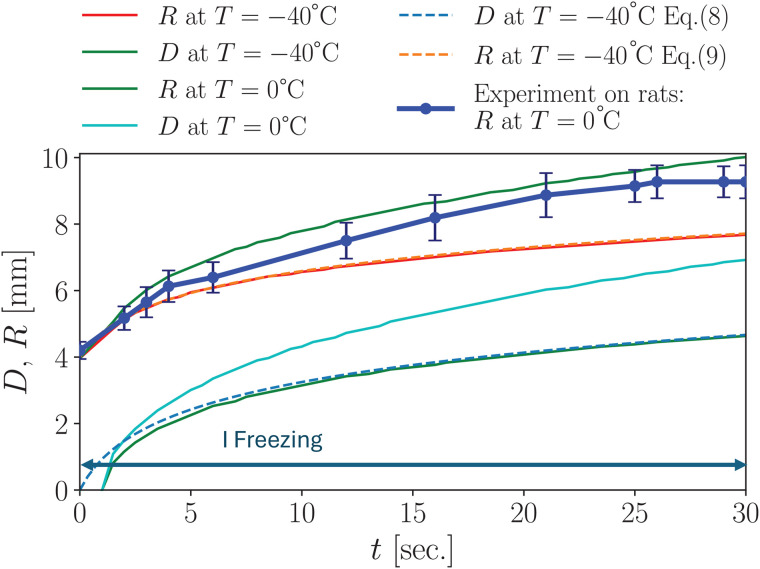
Numerical calculation of the maximum radius *R*(*t*) and maximum depth *D*(*t*) dynamics at point *r* = 0 for two isotherms, 0°C and –40°C during 30 seconds of freezing. Corresponding to stage “I” of the cryoapplication described in Section 3.1.1. The results show strong agreement with previous experimental data on rats (blue curve with dots) for details see Refs. [16,37]. A comparison is also presented with the phenomenological formulas (8) and (9) (dashed lines), representing the depth and radius of the *T* = –40°C isotherm.

Using the parameters defined in Eqs. (33, 34, 35) for a 5% gelatin hydrogel, we performed simulations in cylindrical geometry based on Eq. (23), applying Eq. (36), as Fig 2 illustrates. The heat equation was solved using the finite-difference method.

Fig 2 presents the results of our numerical calculations for hydrogel parameters in a geometry like that used in Refs. [16,37], with a 4 mm radius cryoapplicator pressed into the sample. These simulations allow us to calculate the temperature distribution in depth and analyze the dynamics of various isotherms—particularly the −40°C isotherm, which is considered lethal for living cells, including malignant tumor cells.

Also, thermal imaging is as a valuable tool for calibrating numerical simulations by providing reference points within the surface temperature field. To this end, we compare our computational plots with those obtained via thermal imaging in studies in rats [16,37], represented by the blue curve with dots in Fig 2. As shown in Fig 2 despite the differences in thermodynamic properties between rat tissue and hydrogel (see Appendix B for detailed parameters), the overall surface dynamics of the 0°C isotherm are remarkably similar between our hydrogel-based calculations and the experimental data from rats [16,37]. This similarity can be described by the relative closeness of the thermodynamic parameters and short freezing time (30 sec.). This does not provide sufficient time for the metabolic heat to affect the temperature spread on the skin surface. We investigate the issues caused by each of the typical stages described in Refs. [16,37].

#### 4.1.1 First stage: Freezing in hydrogel.

At this stage, there was used the cryoapplicator, initiating the growth of the ice spot. Over time, this growth slows due to the increasing contact area with warmer hydrogel layers, which reduces the temperature gradient and, consequently, the cooling rate per surface unit.

In our study of hydrogel freezing, we found that the expansion of the ice spot radius—as well as the progression of isotherms such as (*T* = –40°C) can be accurately approximated using a natural logarithmic function. For instance, the depth of the *T* = –40°C isotherm shown in Fig 2 can be described by:


$$D_{-40}(t)=1.303\ln(t+1),$$ (8)

where *t* is time in seconds and $D_{-40}(t)$ is the depth in millimeters. Similarly, the radius of the *T* = –40°C isotherm, is given by:


$$R_{-40}(t)=4+1.03\ln(t+1),$$ (9)

where 4 mm corresponds to the cryoapplicator radius.

#### 4.1.2 Second stage: Fast decrease of frozen spot radius during beginning of thawing.

During the thawing, the cryoapplicator is removed, eliminating the cold source and allowing the ice spot to shrink due to heat flow from the deeper layers of the sample and surrounding material, as defined by the boundary conditions in Eq. (7).

**Fig 3:**
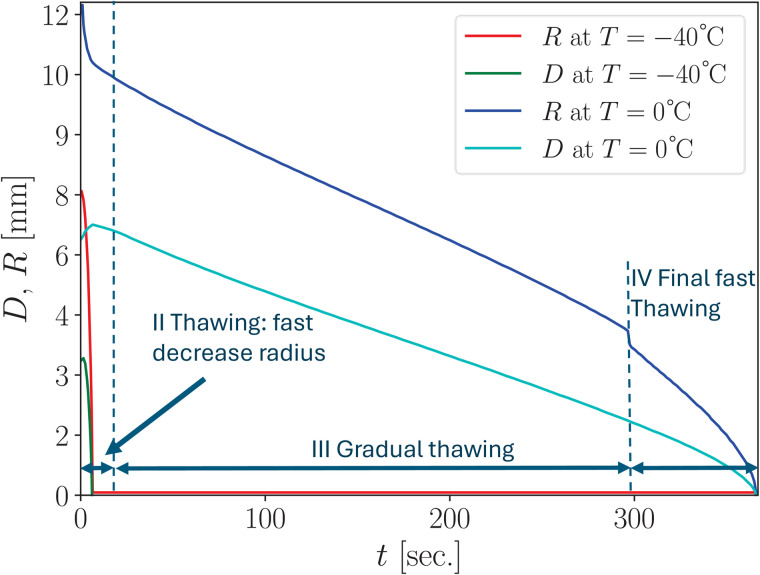
Numerical simulation of the thawing stages showing the dynamics of maximum radius *R*(*t*) and maximum depth *D*(*t*) at point *r* = 0 for two isotherms, 0°C and –40°C. The graph represents calculations following an initial freezing period of 60 seconds.

In real-world conditions, heat is also transferred through the air, and this transfer can vary significantly depending on environmental factors. However, since heat conduction through air is typically much lower than through solid materials, it is neglected in our thawing stage simulations. The dynamics of the frozen front position during thawing are illustrated in Fig 3.

The rapid reduction in the radius of the ice spot is attributed to additional heat transfer through air from the non-insulated sidewalls of the cryoapplicator during the freezing stage. This results in the formation of a thin layer of frozen tissue surrounding the main ice spot, which melts quickly at the thawing onset.

**Fig 4:**
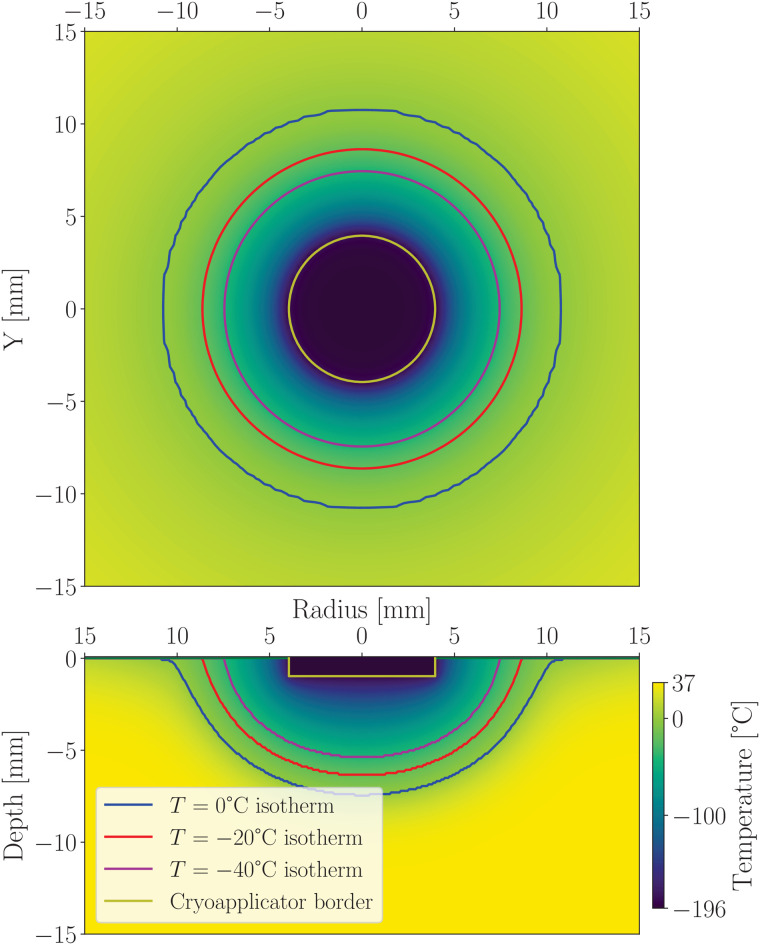
The upper panel displays the surface temperature field after 30 seconds of freezing. The lower panel illustrates the temperature distribution in depth. Several isotherm lines are shown: blue for $T=0^{\circ }\text{C}=T_{\text{f}}$ (upper limit of the phase-change temperature range), red for *T* = –20°C (lower limit of the phase-change range), and violet for *T* = –40°C (lethal temperature for cells). The cryoapplicator boundary is indicated by the yellow line. A frozen ring is formed around the main body of the ice spot. Heat transfer through air is included in the simulation, revealing a thin layer of frozen hydrogel surrounding the central ice region along the zero-temperature isotherm in the bottom panel upper sides. In the upper panel, shown in cylindrical geometry with polar angle symmetry, the thermal image consists of concentric circles representing varying temperatures. Comparison of the top and bottom panels demonstrates how the surface temperature field extends into the sample depth.

To incorporate this effect into the simulation, we used effective thermal parameters for air, as detailed in Appendix A.5. This approach led to the formation of a thin frozen ring on the sample surface encircling the main ice spot, as shown in the bottom panel of Fig 4.

#### 4.1.3 Third stage: gradual thawing.

During this stage, the ice spot thaws slowly, progressing inward toward the center of the cryoapplicator, as illustrated in Fig 3. The thawing process is relatively slow, and the ice spot radius decreases gradually due to the high latent heat of melting. Additionally, the temperature within the ice spot changes gradually due to the distribution of latent heat across the temperature range between the initial melting point *T*_m_ and the cryoscopic temperature *T*_f_. Moreover, the thermal conductivity of frozen tissue is notably higher than that of thawed tissue, resulting in a minimal temperature gradient inside the ice region.

#### 4.1.4 Fourth stage: Final fast decreasing of radius.

At this stage, the radius of the ice spot decreases rapidly due to the cryoapplicator having been pressed a few millimeters into the sample during the freezing phase. As the radius becomes smaller, the rate of decrease accelerates. This effect is driven by the volume difference between thawed and frozen hydrogels at the freezing front, which becomes more pronounced as the radius approaches zero. Specifically, the ratio $\frac{r+\Delta r}{r}$ increases as r→0, while the heat flux remains constant. This results in a relatively small radius and heat transfer surface, amplifying the rate of thawing.

At the onset of this stage, the ice spot beneath the cryoapplicator is thinner than in the surrounding area due to the applicator being pressed a few millimeters into the sample during freezing. As the radius of the frozen hydrogel approaches that of the cryoapplicator, the reduction in ice spot radius accelerates, driven by the thinner ice layer directly beneath the applicator.

### 4.2 Frozen-zone dynamics in biological tissue

**Fig 5:**
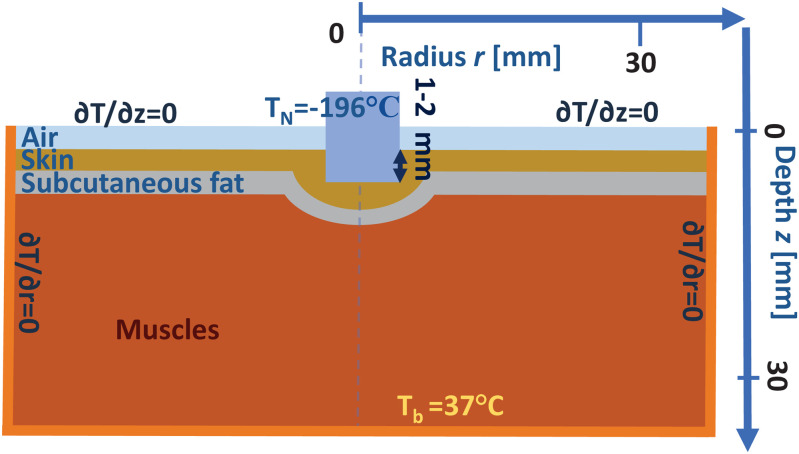
Material configuration used in the cryoapplication simulation for biological tissues. The diagram includes the following components: muscles (orange), subcutaneous fat (gray), skin (brown), cryoapplicator (blue rectangle), and surrounding air (light blue).

Biological tissues differ from hydrogel in that they are composed of multiple layers, each with distinct thermodynamic properties. When using the cryoapplicator to the skin, three primary layers are involved: the epidermis, subcutaneous fat, and muscle tissue, as illustrated in Fig 5, and described in Ref. [16].

#### 4.2.1 Freezing in biological tissues.

**Fig 6:**
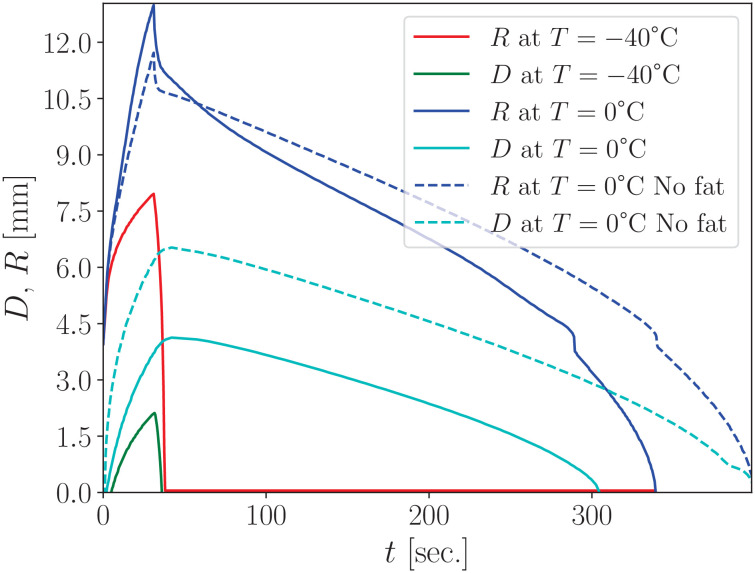
Numerical simulation of the maximum radius *R*(*t*) and maximum depth *D*(*t*) at point *r* = 0 for biological tissues composed of three layers: skin, subcutaneous fat, and muscle. Solid lines represent simulations including all three layers, while dashed lines correspond to simulations excluding the subcutaneous fat layer, based on a 30-second freezing duration. The dynamics are shown for two isotherms 0°C and –40°C. The most pronounced difference arises from the subcutaneous fat layer, which has significantly lower thermal conductivity and therefore slows the depth-wise spread of the ice spot. In the simulation, skin thickness is set to 0.6 mm, fat thickness to 0.3 mm for the solid lines, and 0 mm for the dashed lines.

**Fig 7:**
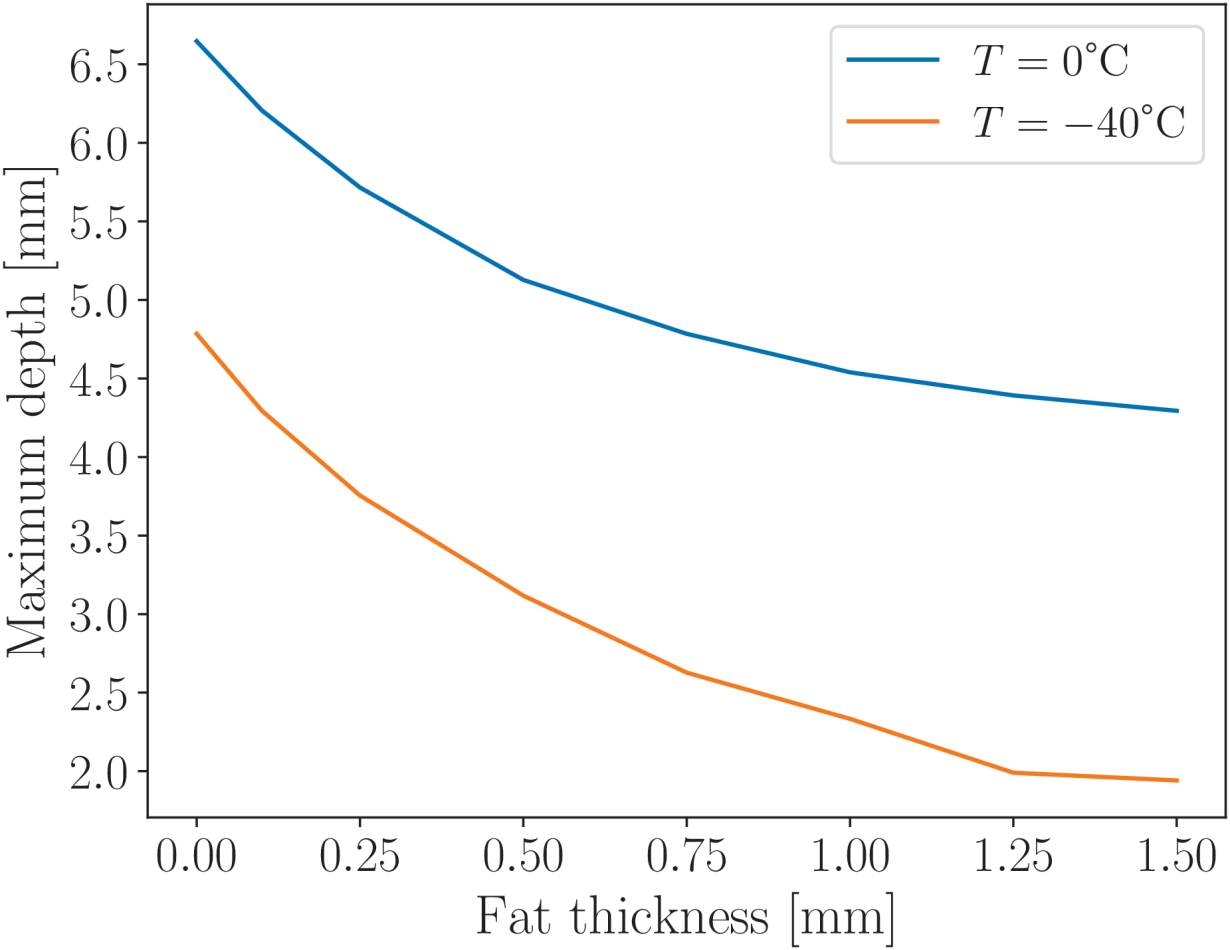
Numerical simulation of the maximum depth reached at point *r* = 0 after 30 seconds of freezing, for varying thicknesses of subcutaneous fat in biological tissues. The model assumes a three-layer configuration with a 0.5 mm skin layer, variable fat thickness, and muscle tissue beneath. The results demonstrate that increasing the thickness of the subcutaneous fat layer leads to a reduction in the maximum depth of the ice spot, due to the low thermal conductivity of fat. This explains the differences in thawing times observed in Fig 6: smaller frozen depth results in faster thawing. Consequently, applying a cryoapplicator to target tissues beneath or within a thick fat layer becomes more challenging and requires significantly longer freezing durations. This effect is especially pronounced at lower temperatures, where the thermal conductivity of fat is substantially reduced, as shown in Fig 11.

The dynamics of the frozen zone in biological tissues are illustrated in Fig 6. Due to the multilayered structure of these tissues, variations in freezing speed were observed. Notably, significant changes occur when layers with markedly different thermodynamic properties are involved. Subcutaneous fat plays a critical role in the depth-wise spread of the ice spot, as shown in Fig 7. This effect is attributed to the substantially lower thermal conductivity of subcutaneous fat, particularly at reduced temperatures. Its conductivity is several times lower than that of other tissue types, as demonstrated in Fig 11. An increased thickness of the fat layer results in a reduction in the maximum depth of the ice spot, further confirming the insulating effect of subcutaneous fat as shown in Fig 7.

#### 4.2.2 Thawing in biological tissues.

Thawing dynamics in biological tissues can qualitatively resemble those observed in hydrogel, especially when the involved tissues—such as skin and muscle—have similar thermodynamic properties. However, when a layer of subcutaneous fat is involved in the model, it begins to slow the thawing process once the frozen zone reaches the fat layer. This effect is evident in Fig 6 from 300 to 350 seconds, where the depth *D* at *T* = 0°C (cyan solid line) shows a noticeable slowing down in thawing as the frozen front encounters the subcutaneous fat. The dashed line in Fig 6, which corresponds to the case with zero subcutaneous fat thickness, exhibits an almost complete absence of the horizontal segment seen in the solid line. This behavior is explained by the thermal conductivity of fat being several times lower than that of other tissues, particularly at reduced temperatures, as illustrated in Fig 11. Although the presence of fat slows down the thawing process, the overall thawing time is shorter in the case with fat. This is because the fat layer limits the depth of the frozen zone, resulting in a thinner ice spot, as shown in Fig 7.

#### 4.2.3 Surface thermal field imaging.

Due to experimental limitations of thermal imaging techniques, only the skin surface can be directly observed. However, our numerical simulations allow visualization of the thermal field at any depth or orientation within the tissue, as demonstrated in Fig 4. Therefore, the surface temperature thermal image presented in Fig 4 upper panel provides data that is consistent with experimental thermal imaging results reported in Refs. [16,17,21].

### 4.3 Experimental frozen-zone dynamics in hydrogel

**Fig 8:**
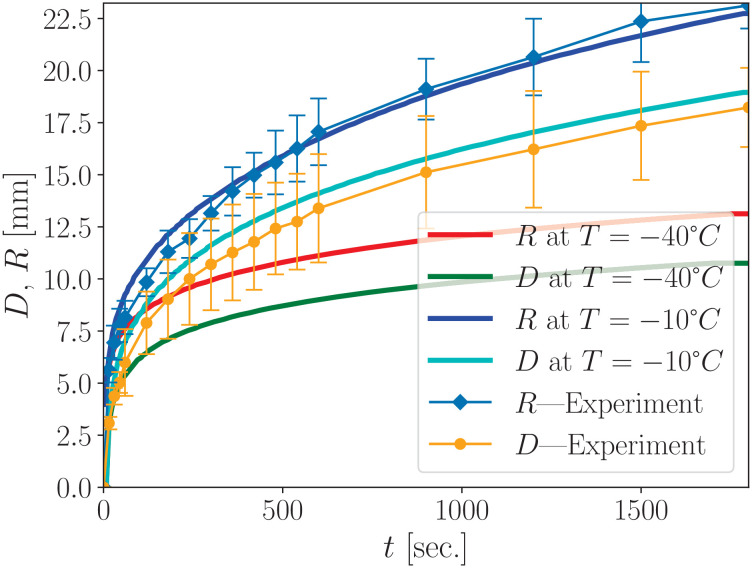
Formation of an ice hemiellipsoid in hydrogel under a cryoapplicator. The experimental dependence of the radius *R*(*t*) and depth *D*(*t*) of the ice hemiellipsoid over time, estimated via visual morphometry, is represented by square symbols. Numerical simulations show the corresponding radius and depth for the isotherm at *T*_ice_ = –10°C reflecting the temperature at which hydrogel transparency visibly changes. These results are comparable to those in Fig 2 but correspond to a longer cryoapplication period and a different isotherm.

The main results of our numerical simulations align well with experimental observations of ice hemiellipsoid formation in hydrogel, as shown in Fig 8. For comparison, we selected the isotherm *T* = –10°C since the hydrogel exhibits a visible change in transparency at this temperature—unlike water, which transitions at 0°C This choice was based on the observation that the experimentally visible radius of the frozen spot was smaller than the radius corresponding to the 0°C isotherm recorded by the thermal imaging camera.

The dynamics of the ice hemiellipsoid radius and depth, as shown in Fig 8, exhibit similar behavior characterized by a rapid increase during the first ten minutes of cryoapplication. To highlight the pronounced growth dynamics, the radius of the ice hemiellipsoid reached 6 mm after just 15 seconds of cryoapplication and doubled within the following three minutes. In contrast, the increase during the ten-minute interval starting from the minute 20 was only 3 mm. After 30 minutes, the radius reached 20 mm. The depth-wise expansion of the ice hemiellipsoid lagged behind its radial growth. At the 30-minute mark, the maximum measured depth was 17.8 mm, yielding a depth-to-radius ratio of 0.79. These results indicate that cryoapplication to a 5% gelatin hydrogel solution produces a deformed hemiellipsoid with an elliptical shape, where the depth is approximately 20% smaller than the surface radius.

Numerical calculations of the maximum radius *R*(*t*) and maximum depth *D*(*t*) at point *r* = 0, compared with experimental data based on visual transparency changes in the 5% gelatin hydrogel, are also presented in Fig 8. Numerical calculations fall well within confidence intervals close to the average experimental value, so the experimental data and numerical calculations agreed well. The dynamics of maximum depth and radius are shown for two isotherms: –10°C, which closely aligns with experimental data due to the transparency change in hydrogel occurring within the phase-change temperature range between *T*_m_ and *T*_f_), and –40°C, representing a deeper thermal threshold. Minor discrepancies between the numerical simulations and experimental results may arise from factors not accounted for in our simplified heat transfer model. These include frost formation on the cryoapplicator sidewalls, influence of external heat sources, and potential inaccuracies in the thermodynamic parameter formulations for hydrogel—particularly at cryogenic temperatures—as well as limitations in experimental measurement precision. Nevertheless, the qualitative behavior remains consistent, primarily driven by the decreasing temperature gradient, which results in a logarithmic-like dependence of both the ice spot radius and depth.

## 5 Discussion

A mathematical model can be a valuable tool for predicting the size and shape of specific isotherms within a controlled environment simulating biological tissues. However, in experimental settings, numerous factors can influence thermal dynamics, potentially leading to discrepancies. Despite these variations, the overall qualitative trends in thermal behavior tend to remain consistent.

### 5.1 Is gel good for describing freezing/thawing of living tissue?

Hydrogel serves as a suitable model medium for simulating cryoapplication in relatively uniform biological tissues [8]. However, it exhibits distinct behavior when compared to layered biological tissues with markedly different thermal properties. One of the key advantages of hydrogels is their well-characterized and consistent thermal behavior, in contrast to living tissues, which display variable thermal properties due to biological heterogeneity.

Typically, hydrogel requires a lower initial temperature—around 20°C—compared to biological tissues, owing to its gel-sol transition occurring near 30°C. This temperature difference does not significantly affect freezing dynamics, as the latent heat associated with phase transition is several orders of magnitude greater than the energy required to cool the material to its cryoscopic temperature. We also assume that the freezing process is rapid, rendering the heat generation of living tissue negligible during the freezing stage. While hydrogel-based experiments cannot provide precise quantitative dynamics due to inherent limitations, they offer substantial advantages in terms of simplicity and reproducibility. The qualitative results are quite comparable to those obtained in experiments on rats and align well with our numerical simulations, as demonstrated in Figs 2 and 8.

#### 4.2 Applicability of mathematical model for different systems.

In principle, the mathematical model can be applied to thermodynamic simulations of various multilayered structures without convection, provided that the materials involved undergo freeze-thawing within a defined temperature range and the geometry of the system is specified.

For cryoapplication scenarios, our model is applicable during the freezing stage to any cryoapplicator placed on the free surface of an object with known thermophysical properties. The only required adjustment is the applicator geometric size. However, if freezing is achieved by spraying the surface with liquid nitrogen or by directing cold nitrogen vapors, the model cannot be used without modification. This is because the model assumes that the initial surface temperature of the object equals the temperature of the applicator –196°C, and that this temperature remains constant throughout the freezing stage—corresponding to boundary conditions of the first kind. In contrast, nitrogen spray methods require knowledge of the heat transfer coefficient at the surface, which corresponds to boundary conditions of the third kind. This coefficient depends on several factors, including jet velocity, the diameter of the jet spread across the surface, and the boiling regime of nitrogen on the surface (film or nucleate boiling), where heat transfer coefficients can vary by several orders of magnitude. Importantly, the model is broadly applicable to thawing scenarios, provided that the geometry and temperature distribution of the system are known.

### 5.3 How can surface termograms be used to conclude on about what’s inside?

Thermal imaging is a valuable tool for calibrating numerical simulations by providing reference points on the surface temperature field. This approach was effectively demonstrated in Figs 4 and 8. In particular, we observed that the frozen zone exhibits an elliptical shape, with the surface radius—visualized via thermal imaging—approximately 20% larger than the depth, which corresponds to the target zone in cryoablation.

### 5.4 Practical hints on using thermal imaging for cryoablation and cryotherapy

By understanding the thermal properties and thicknesses of individual tissue layers—and minimizing temperature leakage through the surrounding air—thermal imaging can be effectively used to estimate the depth of specific isotherms. This is particularly useful when combined with the fast numerical approach described in this study. The structure of biological tissue plays a significant role in shaping thermal distribution and dynamics, as illustrated in Fig 6. These findings are further supported by detailed experimental observations reported in Ref. [21], which align with calculations based on the mathematical model presented here. Accurate knowledge of the thermophysical properties of the target tissue enhances the reliability of thermal imaging as a non-invasive tool for guiding and optimizing cryoablation procedures.

Combining simulation data with thermal imaging of the surface allows real-time prediction of the shape and size of the freezing zone. Continuous monitoring of critical isotherms (–20°C and –40°C) makes it possible to regulate cryo-exposure parameters, ensuring effective destruction of pathological tissues while maintaining the safety of surrounding viable structures.

Thus, the developed mathematical and experimental models of cryo-exposure provide a comprehensive framework for the design of cryosurgical equipment. These models enable the prediction of safe freezing limits, the selection of optimal cryo-exposure durations, and, as a result, an overall increase in the accuracy, safety, and effectiveness of cryosurgical procedures.

## 6 Conclusions

1) We presented a practical and self-contained framework for mathematically modeling thermal distribution in cryoapplicator-based procedures. Using this model, we investigated freezing and thawing dynamics in two distinct media—hydrogel and living tissue—under realistic, temperature-dependent conditions. Our spatio-temporal thermal field calculations were validated against experimental data obtained in gelatin gel [37] and live rat models [16], allowing us to draw the following conclusions: *In vitro* measurements using 5% gelatin hydrogel can serve not only for qualitative assessment but also for quantitative comparison with *in vivo* data.
2) When modeling freeze-thawing in gels, we found that the freezing zone had a hemiellipsoid shape with maximal depth some 20% smaller than the surface radius for the 4 mm cryoapplicator.
3) When modeling freeze–thawing dynamics in living tissue, we found that significant differences in thermodynamic properties between layers—such as the low thermal conductivity of the subcutaneous fat layer—led to deviations from the homogeneous behavior observed in hydrogel models. This may necessitate a substantial adjustment in the duration required to achieve effective cryodestruction.
4) Our findings demonstrate how internal thermal dynamics can be effectively correlated with surface thermal imaging data. The correlation between surface and internal thermal dynamics within the cryoapplication zone enables real-time prediction of the freezing zone’s shape and size, as well as the distribution of critical isotherms at tissue depth during cryosurgery. Although the thermal imager records the temperature distribution only on the surface, continuous visualization of thermal fields during cryoapplication makes it possible to evaluate the dynamics of isotherms (e.g., –20°C and –40°C) that are critical for promised destruction of biological tissues. Monitoring the dynamics of different isotherms opens the possibility of timely changing the parameters of low-temperature exposure in such a way as to minimize the undesirable consequences of excessive cooling of biological tissues, provided that guaranteed cell death in the pathological tissue is achieved.
5) We developed a highly optimized code to perform the heat equation mathematical model with the finite difference method rapidly and efficiently on a modern GPU, and we achieved a significant speedup over the CPU realization.

## A Finite difference method

The finite difference method (FDM) is a numerical technique used to approximate solutions to differential equations [27], such as the heat equation. One of its primary limitations is the potential for stability issues, especially when nonlinear derivatives are involved. To mitigate these challenges, smaller time and spatial steps are typically required. FDM operates on a mesh of discrete points, and its key parameter is the number of these points. This number is determined by dividing the simulated spatial and temporal domain by the spatial step size Δx and time step size Δt respectively.

### A.1 Flat geometry

Consider a mesh with flat geometry. In this configuration, the approximate linearized derivative at each node can be calculated using the right and left finite differences, denoted as $T^{+},T^{-}$, respectively:


$$\frac{\partial T_{i}^{+}}{\partial z}\approx \frac{T_{i+1}-T_{i}}{\left|z_{i+1}-z_{i}\right|},$$ (10)

$$\frac{\partial T_{i}^{-}}{\partial z}\approx \frac{T_{i}-T_{i-1}}{\left|z_{i}-z_{i-1}\right|},$$ (11)

where *z* is the coordinate in the flat geometry. Now, consider the heat equation (1) and compute the total heat change at a single node in the mesh:


$$q_{+}=k\frac{\partial T_{i}^{+}}{\partial z},$$ (12)

$$q_{-}=k\frac{\partial T_{i}^{-}}{\partial z},$$ (13)

$$q=q_{+}-q_{-}=k\left(\frac{T_{i+1}-T_{i}}{\left|z_{i+1}-z_{i}\right|}-\frac{T_{i}-T_{i-1}}{\left|z_{i}-z_{i-1}\right|}\right),$$ (14)

where *k* is the thermal conductivity coefficient, which in realistic scenarios may significantly depend on temperature. Here $q=\frac{dQ}{dS}$ represents the heat flux density, where *Q* is the heat energy and *S* is the surface heat flows. The terms $q_{\pm }$ denote the heat flux in the positive and negative directions, respectively, so the net flux is given by $q=q_{+}+q_{-}$. As for an equidistant flat geometry, where $|z_{i+1}-z_{i}|=|z_{i}-z_{i-1}|=\Delta z$, and with a uniform time mesh $\Delta t=t_{i+1}-t_{i}$, the change in heat flux can be approximated as:


$$\frac{\partial q}{\partial t}\approx k\Delta t\frac{T_{i+1}-2T_{i}+T_{i-1}}{\Delta z}.$$ (15)

This equation becomes exact only in the limit of infinitely small time *dt* and spatial steps *dz*. Therefore, to simplify we consider propagation within each phase, far from the phase-change boundary. In this case, the heat absorbed by a mesh element results solely in a temperature change at that node, with a volume *dV* = *dSdz*. Thus, the finite difference formulation of the heat equation in flat geometry becomes:


$$\frac{\partial T(z,t)}{\partial t}=\frac{kdS\frac{\partial q}{\partial t}}{\rho C_{\text{p}}dV}\approx \;\approx \alpha \Delta t\frac{T_{i+1}-2T_{i}+T_{i-1}}{\Delta z^{2}},$$ (16)

where $\alpha =\frac{k}{\rho C_{\text{p}}}$ is the thermal diffusivity coefficient.

### A.2 Radial geometry

In radial geometry, the derivative is defined in a different way, with the gradient operator given by: $\nabla_{\text{radial}}=\frac{1}{r}\frac{\partial }{\partial r}$. To calculate the derivative accurately between midpoints—which is particularly important in radial geometry—we use a half-shifted radius:


$$r_{+j}=\frac{r_{j+1}-r_{j}}{2},$$ (17)

$$r_{-j}=\frac{r_{j}-r_{j-1}}{2}.$$ (18)

Using these midpoint definitions, the heat flow in radial geometry can be expressed as:


$$q_{+}=k\frac{\partial r_{+}T_{j}^{+}}{r\partial r},$$ (19)

$$q_{-}=k\frac{\partial r_{-}T_{j}^{-}}{r\partial r},$$ (20)

$$q=k\left(\frac{r_{+}T_{j+1}-r_{+}T_{j}}{r_{j}(|r_{j+1}-r_{j}|)}-\frac{r_{-}T_{j}-r_{-}T_{j-1}}{r_{j}(|r_{j}-r_{j-1}|)}\right).$$ (21)

As a result, we obtain the finite difference formulation of the heat equation in radial coordinates:


$$\begin{array}{c} \frac{\partial T(r,t)}{\partial t}=\frac{kdS\frac{\partial q}{\partial t}}{\rho C_{\text{p}}dV}\approx \\ \approx \alpha \Delta t\frac{r_{+}T_{j+1}-(r_{+}+r_{-})T_{j}+r_{-}T_{j-1}}{r_{j}\Delta r^{2}},\; \end{array}$$ (22)

where $\Delta r=r_{j}-r_{j-1}$ is the radial mesh step.

### A.3 Cylindrical geometry

Cylindrical geometry can be modeled by combining flat geometry along the depth coordinate *z*) with radial geometry along the radial coordinate *r*. Assuming polar angle symmetry (i.e., no dependence on the angular coordinate, the heat equation in cylindrical coordinates becomes:


$$\begin{array}{c} T(z_{i},r_{j},t+\Delta t)\approx T_{i,j}\alpha \Delta t\frac{T_{i+1,j}-2T_{i,j}+T_{i-1,j}}{\Delta z^{2}}+\\ +\alpha \Delta t\frac{r_{+}T_{i,j+1}-(r_{+}+r_{-})T_{i,j}+r_{-}T_{i,j-1}}{r_{j}\Delta r^{2}}.\; \end{array}$$ (23)

### A.4 Heat flow transfer for temperature-dependent α(T)

When the thermal diffusivity coefficient *α* depends on temperature, it is essential to maintain continuity of heat flow, assuming no internal heat sources or sinks at specific nodes. This implies that the heat transferred from one node to its neighboring one must equal the heat received by that neighbor. To ensure this, the mutual conductivity between each pair of neighboring nodes must be symmetric. Therefore, instead of using the diffusivity at individual nodes, we calculate an effective thermal diffusivity coefficient for each direction of heat transfer. The updated heat equation becomes:


$$\begin{array}{c} T(z_{i},r_{j},t+\Delta t)\approx \alpha_{+z}\Delta t\frac{T_{i+1,j}-T_{i,j}}{\Delta z^{2}}-\alpha_{-z}\Delta t\frac{T_{i,j}-T_{i-1,j}}{\Delta z^{2}}\\ +\alpha_{+r}r_{+}\Delta t\frac{T_{i,j+1}-T_{i,j}}{r_{j}\Delta r^{2}}-\alpha_{-r}r_{-}\Delta t\frac{T_{i,j}-T_{i,j-1}}{r_{j}\Delta r^{2}}.\; \end{array}$$ (24)

The effective thermal diffusivity coefficients in the axial and radial directions are given by:


$$\alpha_{\pm z}=\left(\frac{2k_{i,j,t}k_{i\pm 1,j,t}}{k_{i,j,t}+k_{i\pm 1,j,t}}\right)\frac{1}{\rho_{i,j,t}C_{\text{p }i,j,t}},$$ (25)

$$\alpha_{\pm r}=\left(\frac{2k_{i,j\pm 1,t}k_{i,j,t}}{k_{i,j,t}+k_{i,j\pm 1,t}}\right)\frac{1}{\rho_{i,j,t}C_{\text{p }i,j,t}},$$ (26)

where *k*_*i*,*j*,*t*_ is the thermal conductivity at node *i*,*j* at time *t*. Here, *i* corresponds to the node index along the *z*-axis, and *j*) corresponds to the node index along the radial coordinate *r*, at time *t*. The analogy of parallel resistors is employed to compute the effective thermal conductivity between neighboring nodes.


$$k_{\text{eff}}=\frac{2k_{1}k_{2}}{k_{1}+k_{2}}.$$ (27)

**Fig 9:**
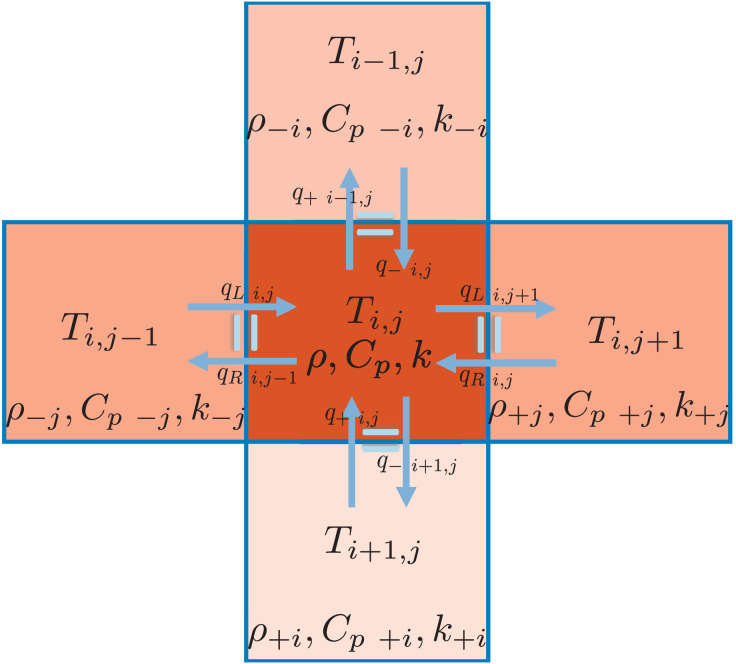
Diagram representation of nodes in the finite difference method (FDM), illustrating heat transfer balance in all directions to the four neighboring nodes. Each node may have a distinct temperature and temperature-dependent properties, including density *ρ*, thermal conductivity *k*, and effective heat capacity *C*_p_. Therefore, neighboring nodes are treated as distinct materials, and the thermal conductivity for the multilayer structure in each direction is calculated using Equation (27).

This formulation is equivalent to modeling consecutive thermal conductivities for two layers, each with thickness 0.5ξ. Here, we assume that half the distance between nodes corresponds to the material and temperature of one node, while the other half corresponds to its neighbor. The parameter *ξ* represents the distance between nodes—either Δr for the radial coordinate or Δz for depth. As equation (27) has illustrated, the distance between the centers of adjacent nodes is *ξ* and within this span, each half corresponds to one of the two neighboring materials. This concept is visually represented in Fig 9.

### A.5 Heat transfer through the air

When modeling thermal conductivity through air, we account for several interacting processes: direct air conduction, convective heat exchange between the cryoapplicator (with non-insulated sides) and the skin, as well as water vapor condensation on both the cryoapplicator and skin surfaces. To incorporate these complex phenomena into the FDM simulation, we apply a series of approximations. If we rely solely on the thermodynamic properties of air—without simulating convection and frost formation—the air layer above the skin rapidly equilibrates to the skin temperature. This limits its cooling effect, as air has significantly lower thermal conductivity and density compared to hydrogel or biological tissue. To address this, we apply effective boundary conditions [38] at the skin surface to capture the combined effects of heat transfer through air. Specifically, we use effective thermal conductivity values [39,40] to simulate temperature distribution across the skin surface and to represent additional cooling via air-mediated heat transfer. We then performed a series of simulations to calibrate these effective parameters by comparing our results with experimental cryoapplication data in rats [16,37] and hydrogel samples [21]. These comparisons revealed an additional radial layer surrounding the primary ice spot, which closely matched the predictions from our model.

## B Parameters

### B.1 The hydrogel parameters

As a model material, we used 5% gelatin gel, with parameters reported in Ref. [8]:


$$\text{initial melting temperature }T_{\text{m}}=-14^{\circ }\text{C},$$ (28)

$$\text{cryoscopic temperature }T_{\text{f}}=-0.1^{\circ }\text{C},$$ (29)

moisture content w=0.95, (30)

$$\text{freezable water fraction }f_{\text{FW}}=0.93,$$ (31)

latent heat of melting of water L=334 kJ/kg, (32)

the temperature-dependent density $\rho [\text{kg}/{\text{m}}^{3}]$:


$$\left\{ \begin{array}{c} \rho_{\text{Gel}}=935.79+8.715\cdot 10^{-2}\cdot T+1.7428\cdot 10^{-4}\cdot T^{2}\\ \text{ for }T\in [-160^{\circ }\text{C},T_{\text{m}}],\\ \rho_{\text{Gel}}=1018.2+16.927\cdot T+1.2044\cdot T^{2}+2.925\cdot 10^{-2}\cdot T^{3}\\ \text{ for }T\in [T_{\text{m}},T_{f}],\\ \rho_{\text{Gel}}=935.79+8.715\cdot 10^{-2}\cdot T+1.7428\cdot 10^{-4}\cdot T^{2}\\ \text{ for }T\in [T_{\text{f}},40^{\circ }\text{C}], \end{array}\right.$$ (33)

the temperature-dependent capacity *C*_p_ [J/(kg K)] and temperature-dependent thermal conductivity *k* [W/(m K)]:


$$\left\{ \begin{array}{c} C_{\text{p}}=2.1+7.9\cdot 10^{-3}T+7.1\cdot 10^{-6}T^{2},\\ \text{ for }T\in [-160^{\circ }\text{C},T_{\text{f}}],\\ C_{\text{p}}=3.95+1.25\cdot 10^{-3}\cdot T,\\ \text{ for }T\in [T_{\text{f}},40^{\circ }\text{C}], \end{array}\right.$$ (34)

$$\left\{ \begin{array}{c} k_{\text{Gel}}=2.191-0.0118\cdot T-6.57\cdot 10^{-5}\cdot T^{2},\\ \text{ for }T\in [-160^{\circ }\text{C},-2.8^{\circ }\text{C}],\\ k_{\text{Gel}}=0.615+0.00181\cdot T,\\ \text{ for }T\in [-2.8^{\circ }\text{C},40^{\circ }\text{C}]. \end{array}\right.$$ (35)

A key feature of freezing in solutions is the change in water concentration within the unfrozen portion. Since ice preferentially forms from pure water, the remaining liquid becomes increasingly concentrated with solutes, resulting in a progressive decrease in its freezing temperature. This phenomenon is particularly relevant in hydrogel and biological tissues.

To account for the latent heat associated with the phase transition, we introduce an effective thermal capacity [41] defined over the temperature range between the initial melting point *T*_m_ and the cryoscopic temperature *T*_f_:


$$\begin{array}{c} C_{\text{p}}^{\text{eff}}=C_{\text{p}}(T)+\left|\Delta H\frac{dw(T)}{dt}\right|=\\ =C_{\text{p}}(T)+Lf_{\text{FW}}w\left|\frac{T_{\text{f}}}{T^{2}}-\frac{T_{\text{f}}}{T_{\text{m}}(T_{\text{m}}-T_{\text{f}})}\right|,\; \end{array}$$ (36)

where *L* is latent heat of water freezing, *f*_FW_ is freezable water fraction, *w* is moisture content, and all of them combined *Lf*_FW_*w* give effective latent heat of freezing.

### B.2 Parameters of biological tissues

In this section, we present the thermodynamic parameters relevant to biological tissues. All such parameters are temperature-dependent; however, available information is limited due to the variability and inconsistency of values across different samples. This variability arises from a wide range of influencing factors, among which hydration—the moisture content level within the tissue—is one of the most significant. Hydration directly affects thermal properties such as heat capacity, thermal conductivity, and phase transition behavior, making it a critical parameter in modeling biological systems.

From Table 1 it is evident that the temperature ranges associated with phase transitions in skin and muscle tissues are quite similar. In contrast, the parameters for subcutaneous fat differ significantly, primarily due to its lower moisture content.

**Table 1:** The thermodynamic parameters listed in Table 1 are representative of mammalian tissues, as values for the same tissue types tend to be consistent across species. These data are compiled from Refs. [30–32].

Tissue	*L* [kJ/kg]	*T*_f_ [°C]	*T*_m_ [°C]
Skin	230	−0.53	−9.15
Subcutaneous Fat	100	−5	−10
Muscle	250	−0.91	−10

**Fig 10:**
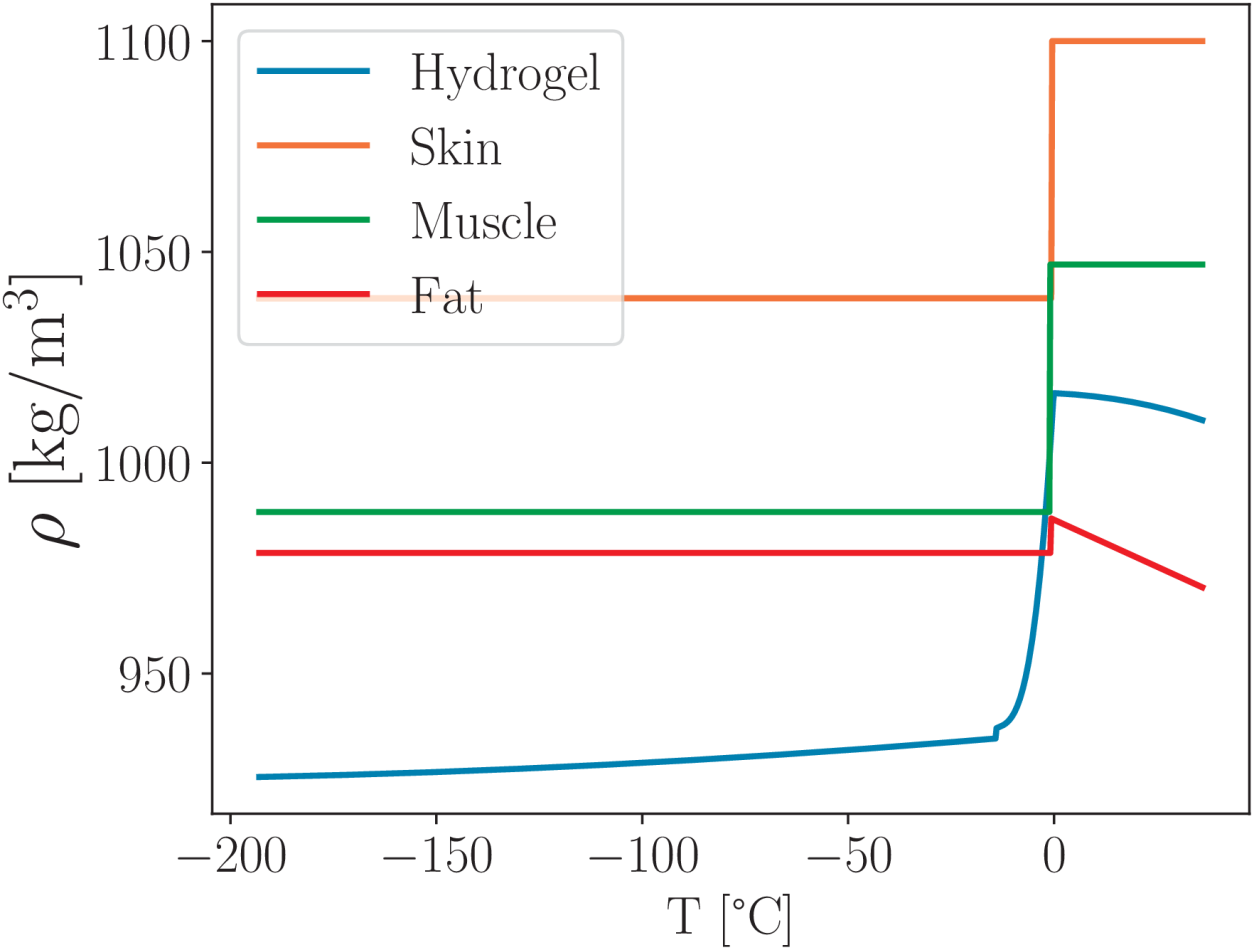
Temperature dependence of the density of biological tissues and hydrogel plotted using data from Table 2 and the temperature-dependent density equations defined in Eq. (33).

In Table 2 and Fig 10 we can see that the density of all biological tissues is quite similar across a wide temperature range.

**Table 2:** Density of the biological tissues *ρ* [kg/m^3^], sourced from Refs. [33,34], summarizes the temperature-dependent density of various biological tissues.

Tissue	*T* > *T*_f_	*T* < *T*_f_
Skin	1100	1039
Subcutaneous Fat	986.47−0.44503T	978.6
Muscle	1047	988.3

In Table 3, due to differences in experimental methodology and data fitting techniques, the thermal conductivity is expressed using distinct polynomial approximations across sources. These variations reflect the complexity of biological tissues and the challenges in standardizing thermal measurements. Nonetheless, the provided formulas offer reliable estimates within the relevant temperature ranges for modeling heat transfer in biological systems.

**Table 3:** Thermal conductivity for the biological tissues *k* [W/(m K)], presents the thermal conductivity values for various biological tissues, collected from Refs. [33,35,36].

Tissue	*T* > *T*_f_	*T* < *T*_f_
Skin	0.424	$1.72+0.0046(-T{)}^{1.156}$
Subcutaneous Fat	$\begin{array}{c} 0.223-5.1\cdot 10^{-5}\cdot \cdot (T-T_{\text{f}})\; \end{array}$	$0.223-3.06\cdot 10^{-}4(T-T_{\text{f}})+0.051(1/T-1/T_{\text{f}})$
Muscle	$\begin{array}{c} 0.4838+9.24\cdot 10^{-4}T\; \end{array}$	$0.46791-6.55\;10^{-3}(T+T_{\text{f}})+0.71175(1/T-1/T_{\text{f}})$

**Table 4:** Thermal capacity for the biological tissues $C_{𝐩}$ [J/(kg K)] [30,31].

Tissue	$T>T_{𝐟}$	$T_{\text{m}}<T<T_{\text{f}}$	$T<T_{𝐦}$
Skin	2742.87+13.166T	5.51(T+273)+143	Same
Subcutaneous Fat	2100+4T	6.51(T+273)+153	Same
Muscle	4.1124T+3347.8	$\begin{array}{c} 380402.7/(0.46-T{)}^{2}\; \end{array}$	$\begin{array}{c} -47624.4T/(0.46-T{)}^{2}\; \end{array}$

**Fig 11:**
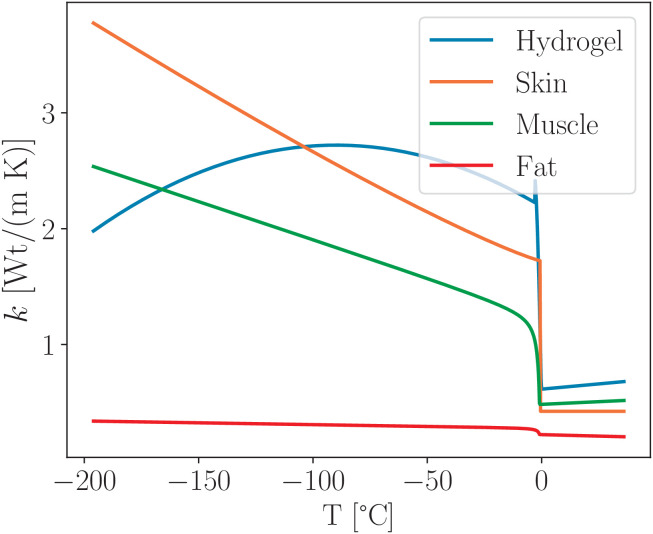
Temperature dependence of the thermal conductivity of the hydrogel and biological tissues constructed using data from Table 3 and the polynomial expressions in Eq. (33).

As shown in Fig 11 the thermal conductivity of subcutaneous fat differs markedly from that of skin and muscle tissues. This discrepancy, primarily due to the lower water content and higher lipid fraction in fat, results in significant alterations to the temperature distribution when subcutaneous fat is incorporated into the computational model. Accurate representation of this variation is essential for realistic thermal simulations in biological systems.

Table 4 presents various formulations for the thermal capacity of biological tissues. For skin and subcutaneous fat, only the standard (sensible) heat capacity is provided. The contribution from latent heat associated with phase change is incorporated using the same expression as Eq. (36) for the hydrogel, substituting $Lf_{\text{FW}}w$, with the appropriate latent heat values from Table 1. In contrast, for muscle tissue, the latent heat component is already embedded within the thermal capacity formulation. Therefore, Table 4 reports the effective thermal capacity for them.

**Fig 12:**
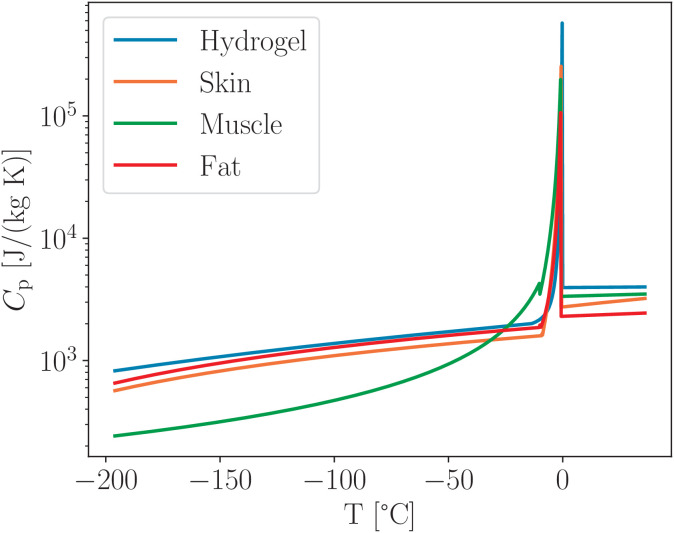
Temperature dependence of the effective thermal capacity for biological tissues and hydrogel, calculated using Eq. (36), which contains latent heat distributed across the phase transition range between *T*_m_ and *T*_f_ for each material.

As illustrated in Fig 12, the maximum value of the effective thermal capacity for subcutaneous fat is comparable to that of other tissues, despite its latent heat being approximately 2.5 times lower. This observation is explained by the narrower temperature range over which phase transition occurs in fat tissue, as shown in Table 1. The total latent heat contribution is computed as the integral of the latent heat over the respective phase change interval for each tissue type.

## C Implementation of the finite-difference method on GPU

To achieve high precision and computational efficiency, we implement the finite-difference method on a Graphics Processing Unit (GPU). GPUs are particularly effective for handling large-scale vector operations, such as those required for solving the two-dimensional cylindrical heat equation. Designed to compute the color of each pixel on a screen, GPUs excel at parallel processing of multidimensional arrays with millions of elements. In our simulation, we leverage this architecture by substituting pixel color calculations with temperature computations, enabling rapid and scalable thermal modeling using optimized numerical algorithms.

### C.1 Some features of solving the heat equation on GPU

By leveraging GPU acceleration with targeted optimizations, we achieved up to a 20,000-fold increase in computational speed compared to CPU-based calculations performed on an Intel i7-11900K processor. Using a high-performance GPU such as the NVIDIA RTX 3080, we were able to perform real-time simulations—sometimes even faster—provided that numerical stability allowed for an increased time step.

Our simulation employs a 512 × 512 node mesh, yielding 262,144 nodes and a spatial resolution of approximately 0.06 mm per node (based on a 3.5 cm domain). This high-resolution grid enables accurate modeling of thin biological layers. The primary constraint in such simulations is the stability of the finite-difference method (FDM) [42], particularly when dealing with temperature-dependent coefficients, as is the case in our study.

To ensure minimal boundary effects, the simulated area was chosen to be approximately 1.3 times larger than the maximum expected size of the frozen region in each dimension. This design minimizes lateral temperature gradients, effectively emulating a semi-infinite medium. Such compact domain sizing is feasible due to the low thermal diffusivity of biological tissues relative to the cryoapplicator high cooling power. The time step is selected to be as large as possible while preserving FDM stability. Typically, we use a 10-microsecond time step, resulting in 100,000 updates per second and over 26 billion temperature updates for a 1-second simulation. In some cases, the algorithm remains stable and precise with time steps up to 300 microseconds, significantly enhancing computational speed. However, the maximum allowable time step is highly sensitive to the thermodynamic properties of the layered materials and may vary even with identical spatial resolution.

### C.2 Optimization strategies for GPU-based heat equation solver

To efficiently handle large arrays in our simulation, we minimized memory copy operations to reduce overhead. Specifically, we implemented the temperature field with (N+2) nodes in each dimension, creating an unused contour around the simulated domain. This design enables straightforward access to neighboring nodes’ temperature and thermal conductivity using shifted indices within the same array.

We maintain a separate array for thermal conductivity to avoid recalculating values for neighboring nodes. These conductivity values are updated per node and synchronized across the mesh, enhancing computational efficiency.

Beyond the stability constraints of the finite-difference method (FDM), the primary performance bottleneck is memory transfer between the CPU and GPU, which is significantly slower than internal GPU memory operations. To mitigate this, we render the temperature field only every 10,000 time steps, reducing unnecessary data movement.

To further improve performance, we simplify frequently used formulas. For example, instead of repeatedly computing the product $C=C_{p}\rho$ for each node, we precompute and analyze the resulting graph to derive a simplified expression for total heat capacity. This avoids redundant calculations and accelerates GPU execution.

The heat equation is solved using the CuPy library in Python, with a custom CUDA raw kernel for single time-step updates. This approach eliminates additional memory copying during kernel invocation, streamlining the computation.

Boundary conditions are handled using two complementary techniques:

- Zero conductivity assignment to nodes outside the computational domain, which ensures no heat transfer beyond the boundaries.
- Boolean masks for directional thermal conductivity, allowing us to suppress heat transfer from the sample to the cryoapplicator to keep its temperature constant while preserving heat flow from the cryoapplicator into the sample.
- Material-specific masks are also used within the kernel to identify and apply appropriate thermophysical properties.

### C.3 Kernel code sample

Below, we present selected components of the CUDA kernel used to compute a single time step in our finite-difference simulation. In the header of the RawKernel, we pass pointers to arrays using the ‘*’ symbol, rather than copying entire arrays during kernel invocation. This approach minimizes memory overhead and improves performance. Additionally, we apply the ‘const’ modifier to input arrays that should remain unchanged within the kernel, ensuring data integrity and preventing unintended modifications.

CalculateFrame_Kernel1 = cp.RawKernel(r”’

extern “C”{

__global__ void CalculateFrame_Kernel1(

float* T, float* K,

const float* rvals,const bool IsGel,

const float* dr,

const int* MaterialMask,

const bool* AlphDownMask,

const bool* AlphUpMask,

const bool* AlphRightMask,

const bool* AlphLeftMask)

{

\\Kernel body

}

}

”’,’CalculateFrame_Kernel1’)

Next, we identify the local indices *i*, *j* as well as global array index *idx* along with the extended indices in the *N* + 2 range. This setup enables the use of shifted coordinates, allowing efficient access to neighboring nodes within the simulation grid. Within this block, we also unpack constants required for computing temperature-dependent thermodynamic parameters.

int id = blockIdx.x * blockDim.x

+ threadIdx.x;

int jd = blockIdx.y * blockDim.y

+ threadIdx.y;

int r_size = int(rvals[0]);

float dtdd = rvals [1];

int i=jd*r_size+id;

int j = i %r_size;

int il=i/r_size+1;

int idx = il * (r_size + 2) + j + 1;

const float rp=rvals[j+6];

const float rm=rvals[j+6+r_size];

const float L_gelCor = rvals [2];

const float Tcor = rvals [3];

const float T_m_Gel = rvals [4];

const float T_f_Gel = rvals [5];

Next, we retrieve the temperature of the current node along with the temperatures of its neighboring nodes and compute their respective differences.

float Tij = T[idx];

const float T_up = T[idx + r_size + 2];

const float T_down = T[idx - r_size - 2];

const float T_right = T[idx + 1];

const float T_left = T[idx - 1];

const float Tij_up = (T_up-Tij);

const float Tij_down = (T_down-Tij);

const float Tij_right = (T_right-Tij);

const float Tij_left = (T_left-Tij);

Next, we calculate the thermal conductivity of the current node by identifying its material type and applying the corresponding temperature-dependent formula. Once updated, we synchronize all GPU threads to ensure that the correct conductivity values are consistently used in subsequent calculations.

float kij = computeGelCond(Tij,T_f_Gel,T_m_Gel,

L_gelCor,Tcor,IsGel,MaterialMask[idx]);

K[idx]=kij;

__syncthreads();

Subsequently, we evaluate the thermal conductivity of the neighboring nodes and compute the heat capacity at each node using the relation $C=C_{\text{p}}\cdot \rho$.

float k_Up =K[idx + r_size + 2];

float k_Down =K[idx - r_size - 2];

float k_Right =K[idx + 1];

float k_Left = K[idx – 1];

float Cij = Heat_Capacity(Tij, T_f_Gel, T_m_Gel,

L_gelCor,Tcor,IsGel,MaterialMask[idx]);

Next, we calculate the effective thermal diffusivity coefficients between the current node and all its neighboring nodes.

float GelAlphaUp =2*(kij*k_Up)/(kij+k_Up)/(Cij);

float GelAlphaDown =2*(kij*k_Down)/(kij+k_Down)/(Cij);

float GelAlphaRight=

2*(kij*k_Right)/(kij + k_Right)/(Cij);

float GelAlphaLeft =2*(kij*k_Left)/(kij+k_Left)/(Cij);

Then, we apply the half-step shifted radius for the radius-related nodes and apply masks to consider the boundary conditions for the cryoapplicator:

float AlphRight = GelAlphaRight*rp

*AlphRightMask[i];

float AlphLeft = GelAlphaLeft*rm;

float AlphUp = GelAlphaUp* AlphUpMask[i];

float AlphDown = GelAlphaDown;

Finally, we calculate the updated temperature field, where edtdd is the precalculated value for time step dt divided by depth step dtdd=dt/dd to avoid redundant calculations and optimize performance.

Tij+= ((Tij_up*AlphUp +Tij_down*AlphDown)*dtdd

+ (Tij_right*AlphRight + Tij_left*AlphLeft));

Finally, we synchronize all GPU threads to ensure that all temperature updates are completed before proceeding.

__syncthreads();

We then assign the newly computed temperature value to the current node with completing the time-step update.

T[idx]=Tij;

As demonstrated, harnessing GPU power for solving the heat equation is conceptually straightforward yet highly effective.

## Supporting information

S1 File Raw data for Fig 2. (XLSX)

S2 File Raw data for Fig 3. (XLSX)

S3 File Raw data for Fig 6. (XLSX)

S4 File Raw data for Fig 7. (XLSX)

S5 File Raw data for Fig 8. (XLSX)
